# High-Throughput Screening of Industrial Brewing Yeast with Lower Synthetic Level of Acetaldehyde During Beer Production

**DOI:** 10.3390/foods14213762

**Published:** 2025-11-02

**Authors:** Shuangxin Han, Kecheng Sun, Xiaoping Hou, Xiujuan Wan, Jiahui Ding, Jianghua Li, Jian Chen, Guocheng Du, Xinrui Zhao, Hua Yin

**Affiliations:** 1 State Key Laboratory of Biological Fermentation Engineering of Beer, Tsingtao Brewery Co., Ltd., No. 56 Dengzhou Road, Dengzhou Road Subdistrict, Shibei District, Qingdao 266023, China; 2 Science Center for Future Foods, Jiangnan University, 1800 Lihu Road, Wuxi 214122, China; 3 Key Laboratory of Industrial Biotechnology, Ministry of Education, School of Biotechnology, Jiangnan University, 1800 Lihu Road, Wuxi 214122, China; 4 Key Laboratory of Carbohydrate Chemistry and Biotechnology, Ministry of Education, Jiangnan University, 1800 Lihu Road, Wuxi 214122, China; 5 Jiangsu Province Engineering Research Center of Food Synthetic Biotechnology, Jiangnan University, 1800 Lihu Road, Wuxi 214122, China; 6 Engineering Research Center of Ministry of Education on Food Synthetic Biotechnology, Jiangnan University, 1800 Lihu Road, Wuxi 214122, China

**Keywords:** high-throughput screening, acetaldehyde, industrial yeast, beer, Co^60^γ mutagenesis, adaptive evolution

## Abstract

The high level of acetaldehyde produced by yeast is a significant concern for all enterprises of beer production. To obtain industrial beer yeast strains with low ability to produce acetaldehyde, a multi-step screening strategy was established, using Co^60^γ mutagenesis, high-throughput screening, and adaptive evolution. A mutant strain (Lager-C) with low production of acetaldehyde was obtained, which had 54% less activity of alcohol dehydrogenase and 64% more activity of acetaldehyde dehydrogenase. Consequently, the formation of acetaldehyde by the Lager-C strain was 63% lower than that of wild-type Lager yeast. In addition, the Lager-C strain maintained phenotypic stability and a consistently lower content of acetaldehyde when continuously fermented for five generations. Furthermore, this mutant strain has similar fermentation performance to that of the wild-type strain. Thus, this novel applied screening strategy and the Lager-C strain will lay a solid foundation for the subsequent development of improved yeast strains for the beer industry.

## 1. Introduction

Beer is one of the most popular alcoholic beverages worldwide, and there is strong consumer demand for higher beer quality. It contains a range of substances that are beneficial to human health, such as soluble fiber, minerals (calcium, iron, magnesium, phosphorus, potassium, zinc, manganese, selenium), and vitamins (group B, A, D, E) [1] Among the many factors that determine the quality of beer, flavor is one of the most important [2]. The characteristic beer flavor mostly consists of metabolites produced by the yeast during the fermentation stage of beer production [3], including ethanol, aroma-active, and off-flavor volatile compounds. Among the off-flavor volatiles, carbonyl compounds have a major influence on the flavor and stability of beer [4]. Among carbonyl compounds, acetaldehyde (ethanal) has the highest content among carbonyl compounds [5], and is a key off-flavor compound with a recognized sensory threshold of 10 mg∙L^−1^ [6], imparting green apple and papery notes. While premium beers contain <3–8 mg∙L^−1^ for a clean profile, levels of 10–25 mg∙L^−1^ yield an immature character, 25–50 mg∙L^−1^ introduce harshness and pungency, and concentrations > 50 mg∙L^−1^ render the beer undrinkable due to extreme irritation [7]. It is also regarded as possibly carcinogenic to humans (IARC Group 2B) [8]. In addition, chemical reactions of aldehydes during beer storage shorten the shelf life of beer. Therefore, reducing the acetaldehyde content should improve the flavor and anti-aging value of beer, which has great potential for industrial production of higher-quality beers. Beyond its flavor attributes, beer also possesses notable health properties.

Conventional control measures to minimize the acetaldehyde content of industrially produced beer are mainly based on process control; however, they are not universally applicable and have limited effectiveness. Therefore, the development of engineered yeast strains is a potentially effective strategy for reducing acetaldehyde biosynthesis by the yeast. For example, Wang et al. employed conventional ultraviolet mutagenesis breeding technology with ethanol as the sole carbon source and 4-MP (4-Methyl pyrazole) as a screening marker, successfully isolating a mutant strain, MA12, with low acetaldehyde production [9]. In another study, Wang and their research team constructed the yeast strain ST31 by deleting the *ADH2* gene and overexpressing the *SOD1* (Superoxide dismutase) and *GSH1* (Glutathione) genes, which reduced acetaldehyde production by 29% compared to the parent strain [10]. However, genetic engineering approaches may alter the profile of other key volatile compounds, and careful management of these metabolic trade-offs is essential for achieving a balanced beer flavor profile. Adaptive laboratory evolution (ALE) improved the performance of *Saccharomyces cerevisiae* for beer production by enhancing microbial tolerance to environmental stressors [11]. However, there are limitations to these strategies, such as clonal interference [12] and the requirement for a relatively long period of mutation treatment [13]. Co^60^γ mutagenesis, a supplementary mutation method, has high lethality but flexible operation and achieves rapid mutation. The mutant *Phaffia rhodozym* strain MK19, developed through combined Co^60^γ mutagenesis and adaptive laboratory evolution, achieved 17-fold higher astaxanthin production compared to the wild-type strain [14]. Therefore, the combination of the above two methods has the potential to achieve rapid and relatively specific mutation.

Another challenge to developing low-acetaldehyde yeast strains is that assay methods for acetaldehyde are time-consuming, laborious, and costly [15], making them unsuitable for high-throughput screening. A colorimetric assay using 96-well plates for high-throughput screening of microorganisms has been developed, but this assay suffers from low accuracy and a high detection limit [16]. Therefore, the development of more efficient assays for acetaldehyde that are suitable for use during beer fermentation and storage is urgently needed to facilitate the construction of high-throughput screening models.

In summary, the high-throughput screening and identification of low acetaldehyde-producing beer yeast strains has great potential to enhance beer flavor and quality and improve food safety. To achieve this goal, we established a multi-step screening platform by integrating Co^60^γ mutagenesis with selective pressure from high acetaldehyde and ethanol-disulfiram, coupled with a high-throughput acetaldehyde assay, to efficiently isolate mutants with low synthesis and high degradation of acetaldehyde. In contrast to conventional single-method approaches, this integrated strategy synergistically combines physical mutagenesis, high-throughput chemical selection, adaptive evolution, and a novel analytical assay to create a more efficient and targeted system for mutant development. Furthermore, the new screening method developed provides a methodological reference for future development of high-performance beer yeasts and metabolic transformation of other industrial microbial strains.

## 2. Materials and Methods

### 2.1. Materials and Chemicals

Acetaldehyde (99.5% GC purity, CAS No. 75-07-0), Shanghai Aladdin Biochemical Technology Co., Ltd. (Shanghai, China); 3-heptanone (99.0% GC purity, CAS No. 106-35-4), Shanghai Macklin Biochemical Co., Ltd. (Shanghai, China); Canadian (Capeland) Pilsner malt (Hopsteiner, Abbotsford, BC, Canada), Magnum pellet hops (Yakima Chief Hops, Yakima, WA, USA) The Cascade pellet hops (with a typical alpha acid content of 4.5–7.0%), and Magnum pellet hops (with a typical alpha acid content of 10.0–14.0%) were commercially sourced imported products.

Acetaldehyde Synthesis Medium (g·L^−1^): Ethanol 10.0, (NH_4_)_2_SO_4_ 5.0, KH_2_PO_4_ 1.0, NaCl 0.1, MgSO_4_·7H_2_O 0.5, CaCl_2_ 0.1, yeast extract 0.1. Sterilized by autoclaving at 115 °C for 20 min.

Acetaldehyde Metabolism Medium (g·L^−1^): NaCl 9.0, acetaldehyde 0.1. Sterilized by autoclaving at 121 °C for 15 min. Acetaldehyde was filter-sterilized separately using a 0.45 μm organic-phase filter membrane and added aseptically to the cooled, sterilized medium.

The beer yeast strain, Lager 497 was obtained from Tsingtao Brewery Co., Ltd., Qingdao, China.

### 2.2. Establishment of a High-Throughput Screening Method Strategy for Yeast Strains with Low Acetaldehyde Production

Co^60^γ mutagenesis was used to produce mutant strains with low acetaldehyde production. Lager 497 was mutated with Co^60^γ during the logarithmic growth phase, and the cells were collected and resuspended to OD600 = 1.5. Bacterial suspension (2 mL) was placed in a 35 mm cell culture dish and irradiated at 0.8 kGy, generating a large mutant library. Mutated bacterial suspension (50 μL, 1 × 10^3^ CFU∙mL^−1^) was spread on resistance-screening agar plates (2.8 g∙L^−1^ acetaldehyde, 10 g∙L^−1^ ethanol, and 0.3 mg∙L^−1^ disulfiram–an aldehyde dehydrogenase inhibitor). After incubation at 30 °C for 3–4 days, the plate surface was rinsed 3–4 times with 1 mL of sterile saline to remove all actively growing mutant strains. The cell density of the recovered cells was adjusted to OD_600_ = 5, and the cells were cultured in adaptive evolution medium (through preliminary gradient experiments, we determined the disulfiram concentration used in the primary screening resistance plates to be 0.3 mg∙L^−1^ and the concentration used in adaptive evolution to be 2.5 mg∙L^−1^) at 30 °C for 2–3 days.

### 2.3. Measurement of Acetaldehyde Content in Mutant Strains with Low Acetaldehyde Production Using 3-Methyl-2-Benzothiazolone Hydrazone

In the presence of ferric chloride, 3-methyl-2-benzothiazolone hydrazone (MBTR) reacts with aldehydes to form an intense blue color. Fermentation supernatant (0.5 mL) and 1 mL MBTR solution (0.4 g of MBTR was dissolved in 100 mL of deionized water) were combined in a 25 mL colorimetric tube and left to stand for 20 min. A total of 1 mL ferric chloride solution (1.0 g of ferric chloride was dissolved in 100 mL of deionized water) was added and left to stand for 10 min, and then deionized water (2.5 mL) was added, and the absorbance was measured at 610 nm.

### 2.4. Simulated Beer Fermentation

Preparation of wort: The wort was prepared by mixing Pilsner malt with water at a ratio of 1:4 (*w*/*v*), at 45 °C. The mixture was then subjected to a stepwise heat treatment: 48 °C for 30 min, 65 °C for 40 min, 72 °C for 10 min, and 78 °C for 10 min (final saccharification). After saccharification, the wort was hot-filtered, boiled for 1 h, and hops were added (0.25% *w*/*v*). The wort was then adjusted to 12 °P. For fermentation, wort (150 mL) was transferred into a 250 mL shake flask and sterilized at 105 °C for 10 min [17].

Strain expansion: The mutant and original strains were inoculated into seed culture medium (10% inoculum) to activate them and maintain high growth rates at low temperatures. The strains were then gradually expanded through three culture stages: 1 mL seed medium at 30 °C, 200 r∙min^−1^ for 12 h; 9 mL YPD at 25 °C, 220 r∙min^−1^ for 12 h; 90 mL YPD at 20 °C, 220 r∙min^−1^ for 12 h. Finally, the temperature-variable expansion was completed in 900 mL YPD at 15 °C, 220 r∙min^−1^ for 12 h.

Shake flask fermentation: The expanded strains were inoculated into the wort (1 × 10^6^ CFU∙ml^−1^) for the pre-fermentation stage; the pre-fermentation was considered finished when the sugar content decreased to 4 °P after 8 days at 12 °C. The post-fermentation was conducted at 4 °C for 7 days.

### 2.5. Determination of Acetaldehyde Content Using Headspace Gas Chromatography

The acetaldehyde content was determined by headspace gas chromatography as described previously [18]. Fermentation medium sample (4.5 mL) and sodium chloride (2 g) were added to a 20 mL headspace sampling vial, and 3-heptanone (0.5 mL, 0.3 mg∙L^−1^) was added as an internal standard.

### 2.6. Determination of Mutant Strain Phenotypic Stability

The 0th-generation strain was obtained by amplifying the strain in YPD medium, and the 1st-generation strain was obtained when the 0th-generation strain was inoculated into fresh acetaldehyde synthesis and metabolism medium (1% inoculum when OD_600_ = 5; OD_600_ = 0.15 after inoculation). The 2nd-generation strain was obtained by amplifying the 1st-generation yeast in YPD medium, and then the 2nd generation was inoculated into fresh acetaldehyde synthesis and metabolism medium to obtain the 3rd generation, and so on for 10 generations. The content of acetaldehyde synthesized and metabolized at different stages by odd-numbered generations of yeast (G1, G3, G5, G7, G9) was determined using a high-throughput spectrophotometric method. Subsequently, the relative deviations in acetaldehyde content within the beer fermentation broth of the same yeast strain across different generations during the fermentation process were compared. Based on these comparisons, the phenotypic stability of the selected yeast strains was verified [19]. However, phenotypic stability within 10 generations is limited, and longer-term studies are required to evaluate genetic stability over extended evolutionary timeframes.

### 2.7. Determination of Fermentation Performance of Different Yeast Strains

Alcohol content: Beer fermentation medium (100 mL) and distilled water (75 mL) were combined in a distillation flask, then distilled into a receiving flask cooled in an ice-water bath, with the volume kept constant during the mixing process. The specific gravity of the distillate was measured at 20 °C using a density bottle, then the alcohol content was calculated based on the specific gravity, as described previously [20].

$$\mathrm{ABV}=x=\frac{Original\;Gravity-Present\;Gravity}{7.6}\times 1000$$

Analysis of volatile compounds: The flavor profile of beer samples was determined by headspace gas chromatography. A mixed standard solution was prepared from acetaldehyde, ethyl acetate, n-propanol, isobutanol, isoamyl acetate, and isoamyl alcohol (all chromatographic grade) dissolved in 4% *v*/*v* aqueous ethanol. The concentration of each component was calculated by the internal standard method. The experimental parameters were as follows: beer sample (4.5 mL) and 3-heptanone internal standard solution (0.5 mL, 0.3 mg·L^−1^) were combined in a 20 mL headspace vial, then the vial was sealed with a crimp septum cap. The concentration of each analyte was determined by comparing the peak area ratio (analyte to internal standard) to the pre-established calibration curve.

The headspace gas chromatography analysis was performed using a GC-2010 system. The separation was achieved with a PEG-20M quartz capillary column (30 m × 0.32 mm I.D.). 3-Heptanone was selected as the internal standard. The headspace sampler conditions were as follows: equilibrium temperature 70 °C, equilibrium time 30 min, transfer line temperature 130 °C, injection time 0.04 min, injector temperature 200 °C, and detector temperature 250 °C. The temperature program was as follows: 40 °C held for 1 min, ramped at 3 °C·min^−1^ to 180 °C, then ramped at 20 °C·min^−1^ to 230 °C and held for 15 min. Carrier gas: 99.99% helium at a flow rate of 1.2 mL·min^−1^. The detector gas flows were nitrogen 30 mL·min^−1^, hydrogen 47 mL·min^−1^, and air 400 mL·min^−1^ [18].

Determination of fermentation rate: The rate was determined by measuring the weight loss from the fermentation, resulting from the loss of carbon dioxide. Specifically, the strains were fermented in 150 mL wort and fermented at 12 °C in a shake flask and weighed regularly; fermentation was stopped when the difference in weight loss between two consecutive days was less than 0.2 g. A weight loss-time curve was plotted to compare the fermentation rates of the different strains [21].

### 2.8. Determination of Biological Characteristics

Growth curve: The activated strain was inoculated into YPD medium (5% inoculum) and cultured at 30 °C and 220 r·min^−1^. Cell growth was determined from the absorbance at 600 nm.

Activity assays for acetaldehyde metabolic enzymes: Yeast cells were washed and resuspended in phosphate buffer (pH 8.0, 0.05 mol∙L^−1^), then the cell walls were disrupted by ultrasonication (treat for 4 s, stop for 5 s, total treatment time 5 min). Crude enzyme solution was obtained by centrifugation at 3600× *g* for 5 min. Different enzymatic reaction systems were selected, and the appropriate amount of cell extract was added. The absorbance of the enzyme/substrate mixture was measured with a microplate reader at 340 nm to calculate the activity of alcohol dehydrogenase I and II, and acetaldehyde dehydrogenase, as described previously [22].

Alcohol Dehydrogenase I

A 250 μL enzyme activity reaction system: 0.05 mol∙L^−1^ glycine-potassium hydroxide buffer solution, 0.001 mol∙L^−1^ NAD^+^, 1 × 10^−4^ mol∙L^−1^ acetaldehyde solution.

Alcohol Dehydrogenase II

A 250 μL enzyme activity reaction system: 0.05 mol∙L^−1^ glycine-potassium hydroxide buffer solution, 0.001 mol∙L^−1^ NADH, 0.1 mol∙L^−1^ ethanol solution.

Aldehyde Dehydrogenase

A 300 μL enzyme activity reaction system: 0.15 mol∙L^−1^ potassium phosphate buffer, 0.001 mol∙L^−1^ NAD^+^, 5 × 10^−4^ mol∙L^−1^ dithiothreitol solution, 1 × 10^−4^ mol∙L^−1^ acetaldehyde solution.

Flocculation rate: The fermented precipitate was washed with 0.01 mol∙L^−1^ EDTA-Na solution and sterile water successively. After centrifugation to remove the supernatant, the fermented precipitate was added to 250 μL of HAC-NAAC buffer solution (pH 4.5), and OD1 was measured at 660 nm. After allowing the mixture to stand at 20 °C for 30 min, 50 μL of the supernatant was obtained from the top of the liquid surface, and the OD2 was measured at 660 nm. Finally, the flocculation F [23] was determined based on the ratio of OD1 to OD2 using HAc-NaAc buffer solution as the blank.

### 2.9. Data and Statistical Analysis

All experiments and measurements were carried out in triplicate. The data were visualized using OriginPro 7.5 software (Origin Lab Corporation, Northampton, MA, USA); one-way ANOVA and Tukey’s range test were used to determine significant differences between measurements, with *p* < 0.05 as the limit for significance.

## 3. Results

### 3.1. Primary Screening of Beer Yeast Mutant Strains for Low Acetaldehyde Production

**Figure 1:**
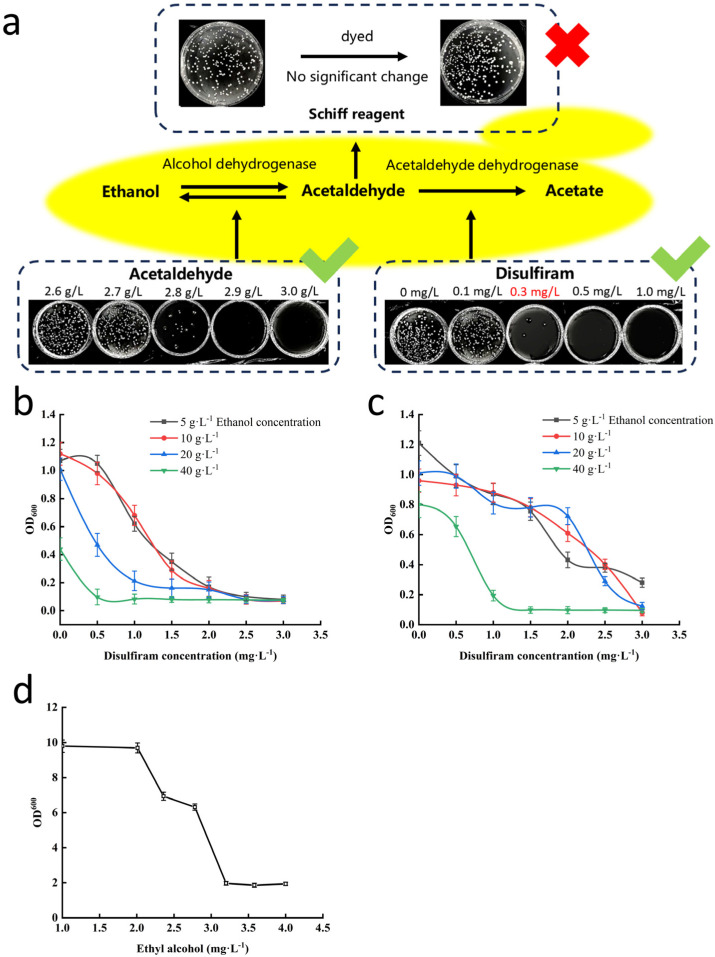
Primary screening process to identify low acetaldehyde-producing beer yeast mutant strains. (**a**) Testing of disulfiram as an inhibitor to screen for industrial yeast mutant strains with low acetaldehyde production; (**b**) growth of yeast in YNB medium containing different concentrations of alcohol and disulfiram (*n* = 3); (**c**) growth of yeast in basic carbon source (2% glucose) medium containing different concentrations of alcohol and disulfiram (*n* = 3); (**d**) growth of yeast in medium with different concentrations of acetaldehyde (*n* = 3).

To screen for low-acetaldehyde yeast strains, the method “single Co^60^-γ mutagenesis combined with disulfiram adaptive evolution” was established (Figure 1a). Many mutants were obtained by treatment with 0.8 kGy of Co^60^-γ radiation, and then the aldehyde dehydrogenase activity was inhibited using different concentrations of disulfiram to inhibit the growth of the yeast mutants and select for strains that could grow normally in a high acetaldehyde environment.

Based on the growth of mutant yeast strains on agar plates, the initial screening conditions were determined as 10 g∙L^−1^ ethanol and 0.3 mg∙L^−1^ disulfiram in a solid basic carbon source medium (containing 2% *w*/*v* glucose; Supplementary Tables S1 and S2). The initial adaptive evolution conditions were 10 g∙L^−1^ ethanol and 2.5 mg∙L^−1^ disulfiram in a basic carbon source (2% glucose) liquid medium (Figure 1a–c).

To enhance the effectiveness of the mutant selection process, mutant yeast strains with low acetaldehyde production were selected for survival on high-concentration acetaldehyde plates mediated by a high capacity for acetaldehyde degradation. The optimal acetaldehyde concentration to select for mutant strains with low acetaldehyde production was determined to be 3.2 g∙L^−1^ (Figure 1d). In total, 107 mutant strains exhibiting robust growth under these inhibitory conditions were obtained (Supplementary Table S3), suggesting they possessed either enhanced acetaldehyde tolerance or degradation capability.

### 3.2. Optimization of Secondary Screening Conditions

The acetaldehyde biosynthetic capacity of mutant strains obtained from primary screening was determined to establish a high-throughput detection method for acetaldehyde production by mutant strains. Currently, a spectrophotometric method is commonly used to measure free acetaldehyde in beer, but it is susceptible to interference and has low accuracy. A chromogenic reagent that can form acetaldehyde derivatives, with high precision and high resistance to solvent interference, such as Schiff’s reagent, potassium nitroferricyanide, or 3-methyl-2-benzothiazolone hydrazine, could overcome this limitation. The best chromogenic reagent for use on beer samples was determined by testing the detection limit, precision, and deviation of each reagent using standard solutions (Table 1). The 3-methyl-2-benzothiazolinone hydrazone spectrophotometric assay had good precision, the lowest detection limit and a distinct color change; therefore, this method was selected for high-throughput screening to compare the acetaldehyde production of beer yeast mutant strains.

The 3-methyl-2-benzothiazolinone assay was used to assess the capacity of mutant strains for the biosynthesis and metabolism of acetaldehyde; the acetaldehyde production of mutant strains with low acetaldehyde biosynthesis was determined in a basic carbon source medium (ethanol instead of glucose). The capacity of mutant strains to metabolize and remove acetaldehyde was determined with acetaldehyde as the carbon source.

**Figure 2:**
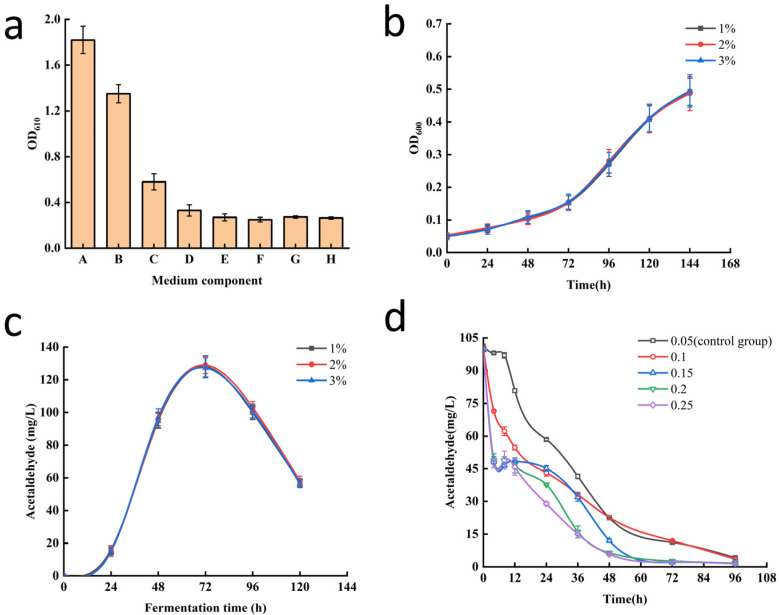
Optimization of secondary screening conditions. (**a**) The influence of different yeast culture media on the chromogenic acetaldehyde assay. A, wort medium; B, YPD medium (containing 2% glucose); C, YNB medium (containing 2% glucose); D, YNB-CAA medium (containing 2% glucose); E, basic carbon source medium (containing 2% glucose); F, basic carbon source medium (containing 5 g∙L^−1^ ethanol); G, 0.02% yeast extract solution; H, 2% glucose solution. (**b**) Impact of inoculation size on fermentation growth of mutant strains. (**c**) Effect of inoculation size on acetaldehyde biosynthesis during fermentation of mutant strains. (**d**) Effect of inoculation size and time on the capacity of mutant strains to metabolize acetaldehyde.

The basic carbon source medium containing 5% ethanol exhibited the least interference with the colorimetric acetaldehyde assay and the growth of beer yeast mutant strains, so it is suitable for the measurement of acetaldehyde content in mutant strains with low acetaldehyde synthesis (Figure 2a). After determining the composition of culture medium, the inoculum size was optimized (Figure 2b,c); however, different inoculation sizes had no effect on yeast growth or acetaldehyde production. To minimize the influence of the inoculated strain on the fermentation, the experiments were carried out with the lowest inoculation (1% *v*/*v*).

To screen for mutant strains with high metabolic activity for acetaldehyde among the initially screened strains, their capacity to degrade acetaldehyde was verified with a standard acetaldehyde solution. Therefore, the acetaldehyde concentration in the standard solution and the initial inoculum size were optimized. The optimal acetaldehyde concentration was 100 mg∙L^−1^, and the initial inoculum size was 0.15 (OD_600_ value). Under these conditions, the volatilization interference of acetaldehyde was relatively small (Supplementary Table S4), and the degradation rate of acetaldehyde in mutant strains was significant during the fermentation (Figure 2d).

### 3.3. Screening of Low-Acetaldehyde Yeast Mutants Obtained by Primary Screening for Low Acetaldehyde Biosynthetic Rate

**Figure 3:**
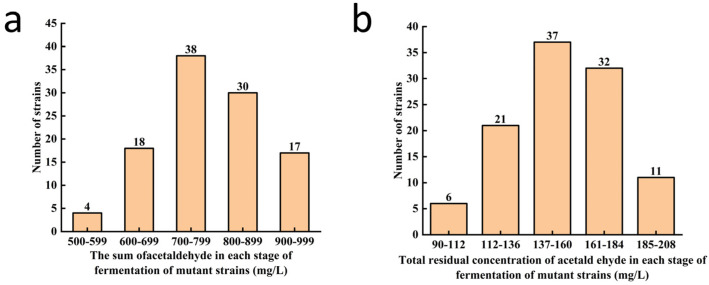
Secondary screening of mutant strains. (**a**) Acetaldehyde production by mutant strains during fermentation. (**b**) Metabolic capacity of acetaldehyde in mutant strains, in terms of residual concentration.

To evaluate the acetaldehyde biosynthesis capacity of the primary screened mutant strains, the total concentration of acetaldehyde in the culture medium was determined using the 3-methyl-2-benzothiazolinone hydrazone spectrophotometric method. Primary screening indicated that most of the 107 mutant strains showed reduced acetaldehyde levels (10.0–51.1%) compared to the initial strain Lager 497 (1011.5 mg·L^−1^, Figure 3a).

The capacity of the primary screened strains to degrade acetaldehyde was evaluated by measuring the concentration of residual acetaldehyde in the culture medium. Of the 107 mutants from primary screening, 27 had lower residual acetaldehyde than Lager 497 (137.4 mg∙L^−1^, Figure 3b). Notably, the residual acetaldehyde of the mutant strain designated Lager-A was 34.2% lower than Lager 497. This observed reduction suggests an improved acetaldehyde degradation capacity; however, potential contributions from factors such as acetaldehyde volatilization or variations in yeast growth rates require further experimental confirmation.

Finally, the spectrophotometric aldehyde assay showed that the capacity of mutant strains to biosynthesize acetaldehyde decreased markedly. Three strains (Lager-A, Lager-B, Lager-C) with low acetaldehyde production, i.e., a decrease of >39.8% compared with Lager 497, were obtained and were used to simulate beer fermentation in shake flasks (Table 2).

### 3.4. Effect of Industrial Yeast Strains with Low Acetaldehyde Production on Beer Fermentation

To test the beneficial effects of mutant strains on beer production, the production capacity of acetaldehyde and differences in biological characteristics between the best re-screened strains (Lager-A, Lager-B, and Lager-C) and the original strain Lager 497 were assessed using a shake-flask simulated beer fermentation.

**Figure 4:**
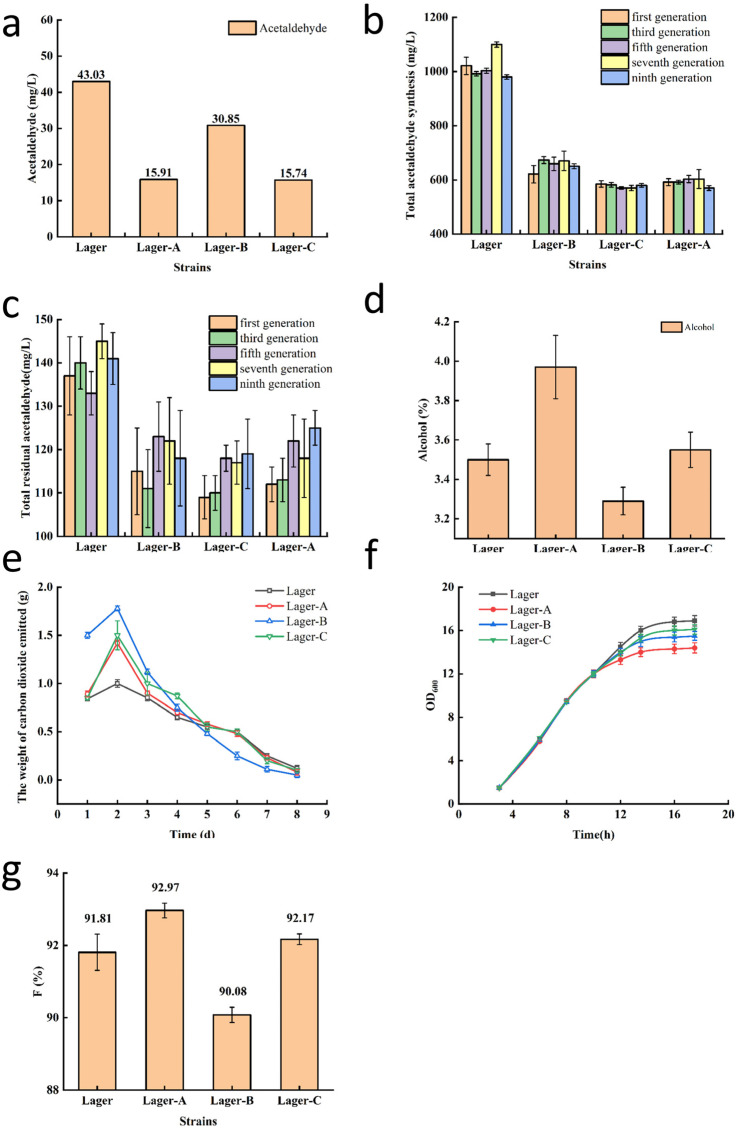
Secondary screening of mutant strains. (**a**) The acetaldehyde concentration of three mutants and Lager497 (*n* = 3). (**b**) The acetaldehyde synthesis content in the fermentation broth of the three mutants and Lager497 (*n* = 3). (**c**) The total residual amount of acetaldehyde metabolism in the fermentation broth of the three mutants and Lager497 (*n* = 3). (**d**) The content of alcohol in the fermentation of Lager497 and mutants. (*n* = 3) (**e**) Rate of fermentation of Lager497 and mutants (*n* = 3). (**f**) Curve of growth of Lager497 and mutants (*n* = 3). (**g**) The flocculability of Lager497 and mutants (*n* = 3).

The simulated beer fermentation produced higher acetaldehyde concentrations than industrial fermentation (Figure 4a); however, the acetaldehyde titer decreased by 63.0 with Lager-A and 63.4% with Lager-C, compared with that of Lager 497. The reduction in acetaldehyde production demonstrates the effectiveness of high-throughput screening. In addition to low acetaldehyde production, strain phenotypic stability is an important factor for the successful industrial application of yeast. To assess the stability of three mutant strains, the acetaldehyde synthesis and metabolic capacity of odd-numbered generations (G1, G3, G5, G7, G9) of the yeast strains was tested; acetaldehyde biosynthesis (Figure 4b) and residual acetaldehyde (Figure 4c; indicates metabolic capacity) were relatively stable over five generations of the mutant strains Lager-A to Lager-C, indicating that the three mutant strains have stable fermentation performance and good genetic stability.

To explore the differences in enzyme activities between low-acetaldehyde yeast and the original strain and their impact on acetaldehyde content, the acetaldehyde metabolic activity of the key enzymes was studied in the original strain and the three mutant strains. The activity of alcohol dehydrogenase I increased by 66.1 and 58.8%, whereas the activity of alcohol dehydrogenase II decreased by 43.8 and 32.3% in Lager-A and Lager-C, respectively, compared with Lager. These changes in enzyme activities in Lager-A and Lager-C would be expected to reduce acetaldehyde production (Table 3).

### 3.5. Fermentation Performance of Low Acetaldehyde Producing Mutant Industrial Yeast Strains Lager-A and Lager-C

Good fermentation performance of beer yeast is essential for efficient beer production, so the fermentation performance of lager-A and -C was determined at a low temperature to assess their brewing characteristics, focusing on alcohol content, flavor, and fermentation rate. The alcohol content significantly increased for Lager-A compared with Lager 497 (Figure 4d).

When screening beer yeast for low acetaldehyde production, it is essential to monitor flavor compounds. The quantification of flavor compounds in the original and mutant strains is presented in Table 4. The total alcohols and total esters increased by 8.8 and 19.4% respectively, and the alcohol-to-ester ratio decreased by 8.9% in Lager-A, which would result in a potential flavor change. In contrast, the variation in alcohol/ester ratios was <3% in Lager-B and Lager-C, indicating no significant difference in flavor.

Mutation of yeast strains may affect the fermentation rate; the fermentation rates of Lager 497 and the mutant strains were similar (Figure 4e). To enhance fermentation performance, the growth profiles and flocculation characteristics of Lager 497 and the mutant strains were determined. The growth of the mutant strains was slightly slower than Lager 497 after 12 h, but the difference was not significant (Figure 4f).

The flocculation rate of beer yeast, after fermentation is completed, has an important influence on the fermentation itself, sedimentation, filtration, yeast recovery, and the final beer flavor. Premature flocculation may lead to delayed or stalled fermentation, whereas excessively slow flocculation can result in turbidity of the beer, generation of off-flavors, and reduced shelf life. The flocculation rate of Lager-B was slightly, but significantly, lower than that of Lager 497, whereas those of Lager-A and Lager-C were slightly, but not significantly, higher (Figure 4g).

In summary, the flavor of beer made with Lager-A was noticeably different from that made with Lager 497, and Lager-C had a flocculation rate closest to that of Lager 497. Among the mutants, Lager-C demonstrated the lowest acetaldehyde synthesis capacity, ideal alterations in enzyme activities, along with a flavor profile and flocculation ability most similar to the original commercial strain.

## 4. Discussion

Although producers and researchers have proposed various measures to control acetaldehyde levels in beer, solutions based on modifying production processes lack universal applicability, while modifying yeast genetic material through molecular approaches still faces challenges in food safety and regulatory compliance, preventing their application in commercial beer production [24]. Single-step mutagenesis is an inefficient method to screen for superior microbial strains [25], stimulating interest in combining conventional breeding methods with mutagenesis. In this study, a novel multi-step screening method was successfully designed, combining Co^60^γ, high-throughput screening (HTS), and adaptive laboratory evolution (ALE), which was efficient. This approach resulted in the mutant strain, Lager-C, with good fermentation performance and low acetaldehyde production.

Co^60^γ mutagenesis causes complex DNA damage and induces high mutation rates in various ways [26], but useful mutants are difficult to obtain by using it alone. ALE was conducted to enhance the adaptability of mutant strains; evolving strains adapt to their environments by altering their metabolic and signaling networks. In this study, 107 strains with improved tolerance were ultimately obtained through long-term adaptive evolution; the tolerance of Lager-C to two inhibitory conditions (YPD medium with 3.2 g∙L^−1^ acetaldehyde and basic carbon source liquid medium with 10 g∙L^−1^ ethanol and 2.5 mg∙L^−1^ disulfiram) was increased by 21.4 and 33.3%, respectively. Therefore, the combination of the two methods proved effective in screening for mutants with stable phenotypic characteristics within 10 generations and was successfully applied to improving industrial brewing yeast.

The accurate quantification of acetaldehyde is crucial for developing low-acetaldehyde beer. While chromatographic methods provide reliable data, they are impractical for high-throughput screening due to their cost and time requirements. Alternative colorimetric assays also present limitations: acetone/vanillin methods lack sensitivity (detection range 50–2000 mg L^−1^), and the 2,4-DNPH method, though more sensitive (linear range 10–500 mg L^−1^ depending on matrix [27]), requires acidic conditions due to poor solubility in neutral solutions, complicating its application. To address these limitations, we selected the 3-methyl-2-benzothiazolinone hydrazone (MBTH) method for its superior characteristics: high precision, broad linear range (0.1–600 mg L^−1^), low detection limit (0.15 mg L^−1^), and straightforward operation without the need for derivatization. These features make it particularly suitable for sensitive determination of acetaldehyde in beer and compatible with high-throughput screening formats. Therefore, we established and optimized an MBTH-based assay to enable efficient screening of mutant yeasts during beer fermentation. Meanwhile, the MBTH assay offers advantages of rapid analysis, low cost, and high-throughput capacity, while demonstrating superior accuracy compared to the other reagents. This makes it a promising tool for rapid screening and process monitoring in industrial settings.

In summary, the Lager-C mutant strain was isolated from a mutant library using a new high-throughput screening strategy. Lager-C exhibited superior low-acetaldehyde production characteristics compared with the parent industrial yeast strain, Lager. The reduction in acetaldehyde content slows down the formation of off-flavors during beer transport and storage, thereby extending the shelf life and enhancing flavor stability. This multi-step screening approach provides a platform for the future development of industrial yeast strains and their potential applications.

## 5. Conclusions

The high level of acetaldehyde in beer remains a major challenge for brewers, as it directly compromises flavor stability. To address this issue, we established a multi-step high-throughput screening strategy integrating Co^60^γ mutagenesis with selective pressure from high acetaldehyde and ethanol-disulfiram, coupled with an efficient MBTH colorimetric assay. This comprehensive approach enabled the rapid identification of mutant strains with enhanced acetaldehyde degradation capability and reduced synthesis. The obtained mutant strain Lager-C exhibited a remarkable 63% reduction in acetaldehyde production, attributed to a 54% decrease in alcohol dehydrogenase activity and a 64% increase in acetaldehyde dehydrogenase activity. Importantly, Lager-C maintained excellent phenotypic stability over multiple generations while showing fermentation performance comparable to the original industrial strain. This work provides both an effective non-GMO solution for controlling acetaldehyde in beer and a scalable screening methodology for developing superior industrial microbial strains.

## Figures and Tables

**Table 1:** The results of acetaldehyde content detected by the spectrophotometric method.

Method	Detection Limit (mg∙L^−1^)	Accuracy (%)	Linearity Range (mg∙L^−1^)	Apparent Color of Detection Limit	Correlation Coefficient	Linear Equation
a	500	0.34–1.8	400–1800	Almost colorless	0.997	y = 15060.9x + 336.3
b	120	0.27–0.62	600–1800	Almost colorless	0.996	y = 3412.6x + 306.5
c	0.15	0.18–0.66	0.1–600	Light blue	0.999	y = 28.92x + 0.73

a: Schiff’s reagent; b: Potassium nitroferricyanide; c: 3-methyl-2-benzothiazolone hydrazine.

**Table 2:** Results of secondary screening for mutant strains with low acetaldehyde production.

Starting Strain	Mutation Method	Screening Plate and Adaptive Evolution Solution	Re-Screening Strategy	Transformation Efficiency (%)	Serial Number
Lager497	^60^Coγ	High concentration of acetaldehyde	Strong metabolism	34.2	A
			Low synthesis	51.1	C
		Ethanol-disulfiram	Strong metabolism	10.3	B
			Low synthesis	43.4	B

Note: The strains with the same serial number are the same strain.

**Table 3:** The key enzymes of acetaldehyde metabolism of Lager 497 and mutant strains (U·mg^−1^, *n* = 3).

Strain	Ethanol Dehydrogenase I Activity	Ethanol Dehydrogenase II Activity	Acetaldehyde Dehydrogenase Activity
Blanks (water)	0.11	0.11	0.17
Lager	1.77	2.01	7.15
Lager-A	2.94	1.13	7.70
Lager-B	1.58	2.98	7.47
Lager-C	2.04	1.36	8.77

**Table 4:** Some indexes with the fermentation of the initial strain and mutants (mg∙L^−1^, *n* = 3).

Strain	Lager	Lager-A	Lager-B	Lager-C
Ethyl acetate	9.16 ± 0.88 ^a^	10.91 ± 0.58 ^c^	8.67 ± 0.39 ^c^	8.03 ± 1.55 ^c^
Isoamyl acetate	0.40 ± 0.02 ^b^	0.50 ± 0.03 ^c^	0.38 ± 0.01 ^c^	0.41 ± 0.03 ^c^
N-propanol	14.15 ± 1.23 ^a^	17.98 ± 0.84 ^c^	13.16 ± 0.63 ^c^	13.44 ± 2.08 ^c^
Isobutanol	14.97 ± 0.89 ^c^	13.89 ± 0.55 ^c^	13.69 ± 0.99 ^c^	11.08 ± 1.18 ^c^
Isoamyl alcohol	57.13 ± 3.89 ^c^	61.96 ± 2.51 ^c^	54.93 ± 4.60 ^c^	53.62 ± 5.46 ^c^
Alcohol	3.5 ± 0.08 ^a^	3.97 ± 0.16 ^c^	3.29 ± 0.07 ^a^	3.55 ± 0.09 ^c^
Rate of change in alcohol ratio (%)	-	−8.85	0.16	2.62

0.01 < ^a^ < 0.05, ^b^ < 0.01, ^c^ > 0.05.

## Data Availability

The original contributions presented in this study are included in the article. Further inquiries can be directed to the corresponding authors.

## Supplementary Materials

The following supporting information can be downloaded at: https://www.mdpi.com/article/10.3390/foods14213762/s1. Supplementary Tables: Supplementary Table S1, Carbon source concentration of ethanol-disulfiram screening medium; Supplementary Table S2, Screening medium inhibition concentration; Supplementary Table S3, Results of the preliminary screening of mutant strains with lower aldehyde production; Supplementary Table S4, Metabolic capacity of mutant strains to acetaldehyde.

## References

[1] Kaczyński P., Iwaniuk P., Hrynko I., Luniewski S., Lozowicka B. (2024). The effect of the multi-stage process of wheat beer brewing on the behavior of pesticides according to their physicochemical properties. Food Control.

[2] Jaeger S.R., Worch T., Phelps T., Jin D., Cardello A.V. (2021). Effects of “craft” vs. “traditional” labels to beer consumers with different flavor preferences: A comprehensive multi-response approach. Food Qual. Prefer..

[3] Kucharczyk K., Tuszyński T., Żyła K., Puchalski C. (2020). The effect of yeast generations on fermentation, maturation and volatile compounds of beer. Czech J. Food Sci..

[4] Reichel S., Carvalho D.O., Santos J.R., Bednar P., Rodrigues J.A., Guido L.F. (2021). Profiling the volatile carbonyl compounds of barley and malt samples using a low-pressure assisted extraction system. Food Control.

[5] Shin K.-S., Lee J.-H. (2019). Acetaldehyde contents and quality characteristics of commercial alcoholic beverages. Food Sci. Biotechnol..

[6] Hernandes K.C., Souza-Silva E.A., Assumpcao C.F. (2020). Carbonyl compounds and furan derivatives with toxic potential evaluated in the brewing stages of craft beer. Food Addit. Contam. Part A-Chem. Anal. Control. Expo. Risk Assess..

[7] Shen N., Wang J., Liu C., Li Y., Li Q. (2014). Domesticating brewing yeast for decreasing acetaldehyde production and improving beer flavor stability. Eur. Food Res. Technol..

[8] Dirk, Lachenmeier W., Sohnius E.-M. (2008). The role of acetaldehyde outside ethanol metabolism in the carcinogenicity of alcoholic beverages: Evidence from a large chemical survey. Food Chem. Toxicol..

[9] Wang J.J., Shen N., Yin H., Liu C., Li Y., Li Q. (2013). Development of industrial brewing yeast with low acetaldehyde production and improved flavor stability. Appl. Biochem. Biotechnol..

[10] Wang Z.Y., Wang J.J., Liu X.F. (2009). Recombinant industrial brewing yeast strains with ADH2 interruption using self-cloning GSH1+CUP1 cassette. FEMS Yeast Res..

[11] Zhang Q., Jin Y.-L., Fang Y., Zhao H. (2019). Adaptive evolution and selection of stress-resistant *Saccharomyces cerevisiae* for very high-gravity bioethanol fermentation. Electron. J. Biotechnol..

[12] LaCroix R.A., Palsson B.O., Feist A.M. (2017). A model for designing adaptive laboratory evolution experiments. Appl. Environ. Microbiol..

[13] Cui L.Y., Wang S.S., Guan C.G., Liang W.F., Xue Z.L., Zhang C., Xing X.H. (2018). Breeding of methanol-tolerant Methylobacterium extorquens AM1 by atmospheric and room temperature plasma mutagenesis combined with adaptive laboratory evolution. Biotechnol. J..

[14] Miao L., Wang Y., Chi S., Yan J., Guan G., Hui B., Li Y. (2010). Reduction of fatty acid flux results in enhancement of astaxanthin synthesis in a mutant strain of Phaffia rhodozyma. J. Ind. Microbiol. Biotechnol..

[15] Tian J. (2010). Determination of several flavours in beer with headspace sampling–gas chromatography. Food Chem..

[16] Gurdo N., Poisson N.G.F., Juárez A.B., de Molina R.M.C., Galvagno M.A. (2018). Improved robustness of an ethanologenic yeast strain through adaptive evolution in acetic acid is associated with its enzymatic antioxidant ability. J. Appl. Microbiol..

[17] Trueba P.B., Jaskula-Goiris B., Ditrych M., Filipowska W., De Brabanter J., De Rouck G., Aerts G., De Cooman L., De Clippeleer J. (2021). Monitoring the evolution of free and cysteinylated aldehydes from malt to fresh and forced aged beer. Food Res. Int..

[18] Hernandes K.C., Souza-Silva É.A., Assumpção C.F., Zini C.A., Welke J.E. (2019). Validation of an analytical method using HS-SPME-GC/MS-SIM to assess the exposure risk to carbonyl compounds and furan derivatives through beer consumption. Food Addit. Contam. Part A.

[19] Lv Y., Gu Y., Xu J., Zhou J., Xu P. (2020). Coupling metabolic addiction with negative autoregulation to improve strain stability and pathway yield. Metab. Eng..

[20] Einfalt D. (2021). Barley-sorghum craft beer production with Saccharomyces cerevisiae, Torulaspora delbrueckii and Metschnikowia pulcherrima yeast strains. Eur. Food Res. Technol..

[21] Oomuro M., Watanabe D., Sugimoto Y., Kato T., Motoyama Y., Watanabe T., Takagi H. (2018). Accumulation of intracellular S-adenosylmethionine increases the fermentation rate of bottom-fermenting brewer’s yeast during high-gravity brewing. J. Biosci. Bioeng..

[22] Sung C., Kim S., Oh C., Yang S., Han B., Mo E. (2012). Taraxerone enhances alcohol oxidation via increases of alcohol dehyderogenase (ADH) and acetaldehyde dehydrogenase (ALDH) activities and gene expressions. Food Chem. Toxicol..

[23] Sariki S.K., Kumawat R., Singh V., Tomar R.S. (2019). Flocculation of *Saccharomyces cerevisiae* is dependent on activation of Slt2 and Rlm1 regulated by the cell wall integrity pathway. Mol. Microbiol..

[24] Yin H., Liu M., Deng Y. (2017). Reduced acetaldehyde production by genome shuffling of an industrial brewing yeast strain. J. Inst. Brew..

[25] Zhu Y., Sun J., Zhu Y., Wang L., Qi B. (2015). Endogenic oxidative stress response contributes to glutathione over-accumulation in mutant Saccharomyces cerevisiae Y518. Appl. Environ. Microbiol..

[26] Akamatsu K., Shikazono N., Saito T. (2017). New method for estimating clustering of DNA lesions induced by physical/chemical mutagens using fluorescence anisotropy. Anal. Biochem..

[27] Kozaeva E., Mol V., Nikel P.I., Nielsen A.T. (2022). High-throughput colorimetric assays optimized for detection of ketones and aldehydes produced by microbial cell factories. Microb. Biotechnol..

