# Supplementary material for: High-Throughput Screening of Industrial Brewing Yeast with Lower Synthetic Level of Acetaldehyde During Beer Production
Source: Foods. 2025 Nov 2;14(21):3762. doi: 10.3390/foods14213762 (PMC12607408; doi:10.3390/foods14213762)
Supplement: Supplementary file 1 [file foods-14-03762-s001.zip › foods-3911632-supplementary.pdf]

Supplementary Table S1 Carbon source concentration of ethanol-disulfiram screening medium

Ethanol concentration（$g \cdot L^{-1}$）	Colony number of basic carbon source medium	YNB colony number of culture medium
5	217	112
10	289	74
20	105	6
40	1	0

Supplementary Table S2 Screening medium inhibition concentration

Disulfiram concentration（$g·L^{-1}$）	Colony number
0	289
0.1	188
0.3	87
0.5	0
1.0	0
2.0	0

Supplementary Table S3 Results of the preliminary screening of mutant strains with lower aldehyde production

Starting strain	Mutation method	Screening plate and domestication medium	Number of primary strains screened	Sum total
Lager497	^{60}Coγ	High concentration acetaldehyde	104	107
		Ethanol-disulfiram	3	

Supplementary Table S4 Metabolic capacity of mutant strains to acetaldehyde

Acetaldehyde standard solution concentration（$mg·L^{-1}$）	12 h margin percentage（%）	24 h margin percentage（%）
20	68.30	44.77
40	69.75	51.43
60	68.02	56.44
80	68.30	53.02
100	80.80	58.51
120	71.70	49.57
140	69.33	49.67
160	68.96	48.66
180	68.03	49.32
200	68.64	50.02
